# Supplementary material for: Inclusion of environmentally themed search terms improves Elastic net regression nowcasts of regional Lyme disease rates
Source: PLoS One. 2022 Mar 10;17(3):e0251165. doi: 10.1371/journal.pone.0251165 (PMC8912246; doi:10.1371/journal.pone.0251165)
Supplement: S1 Table — Terms for each region were identified via Google Correlate using region specific Lyme disease rates from training period data. (PDF) [file pone.0251165.s001.pdf]

S1 Table Search terms identified by Google Correlate from Each Region

Northeast Search Terms Identified by Google Correlate™	Midwest Search Terms Identified by Google Correlate™
necbl, freezing squash, coastal plains league, winchester royals, blues on the mall, valle league baseball, pick your own berries, little league allstars, wilmingtong sharks, swamp bats, vacation bible school songs, bible school songs, berry picking, blueberry farms, water park ga, bend elks, summer recipe, white water six flags, baby toads, vbs games, league allstars, stroke and stride, fresh cherry, swimming parks, water park fresno, brooklyn concerts, free concerts in nyc, falmouth commodores, rose tree park, central park free concerts, picking blueberries, glacier pilots, keene swamp bats, harvesting basil, summer run, wild raspberries, pitting cherries, concerts brooklyn, alabama water park, tomato plant problems, summer sizzler, freedom run, danville dans, symphony in the park, broune braves, pick your own blueberries, blossom rot, movies under the stars, fresh cherry pie, all stars baseball, water park atlanta, whie water oklahoma, movies on the rocks, freezing cherries, free outdoor concerts, little league all stars, free concerts, cherry recipes, duluth huskies, alive at five, collegiate baseball league, squash diseases prospect park bandshell, mankato moondogs, sunset concert, marketfest, newport gulls, staunton braves, lake fest, hays larks, summer in the park, concert in park, freezer jam, cherry pitter, the berry patch, harwich mariners, wilson tobs, church festivals, striped cucumber beetle, alaska baseball league, nycbl, freedom fest, passing league, fourth of july cakes, milwaukee festival, fresh cherry recipes, july calendar, summer concerts in the park, dallas water park, gateman, tomato blossom, harvesting dill, watertown wizards, anchorage bucs, asa metro, akron racers, mallards baseball, colorado 4th of july, big surf, canning squash	music in the park, fried squash recipe, homemade ice cream recipes, coney island cincinnati, beaches in ny, isalnd water park, wild water adventures, harvesting garlic, summer appetizers, sprinkler park, cool river tubing, nyc pools, tubing in helen, wetlands water park, river water park, fried squash, lake beaches, roseville water park, water west, wild water west, festibals milwaukee, harvesting basil, wolf river campground, beaches in ct, family aquatic cetner, blueberry farm, drive in ohio, waterworld concord, illinois river float, blueberry patch, splash country, music under the stars, dollywood splash, nock a mixon, 49 drive in, fort dupont park, movies in the park, dollywood splash country, fantasy lake, splashdown water park, motels in ocean city, hotels near worlds of fun, pikes peak cog railway, venture river, zucchini blossom, kings island discount, cooling bandana, summer desserts, buffalo state park, island water, outdoor water parks, dupont park, waterworld denver, bourne braves, festival colorado, helen tubing, free concerts, beach chicago, easy summer recipes, pick blueberries, centennial beach, the comal river, brooklyn concerts, watermelon recipe, wild water, illinois river float trips, tubing helen, little buffalo state park, funplex omaha, beaches in ohio, dorm bedding, wave country nashville, melody 49 drive in, beaver dam swim club, valley beach, watermelon drinks, ocean beach new london, free concerts nyc, harvesting dill, bay water park, what do baby toads eat, camp nock a mixon, beaches in michigan worlds of fun discount, nyc public pools, sherwood island state park, grilled squash, river float trips, campground colorado, marketfest, crystal springs east brunswick, free outdoor concerts, luverne drive in, hoboken movies under the starts, summer dessert, summer soup, motel wisconsin dells, pool cooler, waterville gulf shores, summer sizzler

Southeast Search Terms Identified by Goggle Correlate™	Southwest Search Terms Identified by Google Correlate™
intex pool cover, potato beetles, top summer songs, free summer movie, martinsville mustangs, ph in pools, swampdogs, good summer reading, intex pool covers, alabama water parks, summer session ii, passing league, fayetteville swampdogs, so cal asa, ultimate camp, alive at five, local swimming pools, hornell dodgers, baking soda pool, summer cakes, coastal plains league, wood bat tournament, clear pool water, npr summer reading, vertical window air conditioner, swim shirts, herndon braves, asheboro copperheads, amc free movies, watertown wizards, amc free, amc summer, mankato moondogs, ohcra, woodchucks baseball, wood bat, springtails, rooftop films, freezing spinach, east cobb baseball, fun days, fun water games, socal asa, nycbl, free summer kids movies, summer treats, bag toss, pool supply stores, good summer books, quick set pool, wilson tobs, baler, baler parts, bend elks baseball, hay rake, haier air, us club, cloudy pool water, intex, valley beach, omaha public shcools, pool rope, summer family fun, get rid of woodchucks, summer movie camp, bend elks, summer books, milky pool water, green pool water, fun summer things, palmetto falls, green pool, diamond devils, pa pool, blue bayou, auburn water park, az water parks, laramie colts, pruning tomato, danville dans, park pools, movies on the rocks, summer things, care of tomato plants, camp resource, intex pumps, banzai water, cloudy pool, summertime songs, cast cover, elite camp, point mallar, wilmington sharks, pool times, summer things to do, blue bayou in baton rouge, summer newsletter, omaha pools, easy camp, alabama water park	bonelli park san dimas, how to make string bracelets, make string bracelets, paco perez, club corolla, moon bay ca, half moon bay ca, ping eye 2 black dot, string bracelets, acushnet river valley golf course, boston ma attractions, pigeon forge hotels, kimball farms westford ma, alaska outdoor, ceiling fans installation, tetherow golf, world rv, recipes on the grill, terrafin sst, cataratas del niagara, solar star attic fan, cave of the winds colorado springs, best roller coaster in the world, dog beach huntington beach, ca water, williamsburg va hotels, free camping sites, carpinteria, paradise lodge mt rainer, pier 45, adirondack extreme adventure, tropical seas myrtle beach, hood river oregon hotels, harbin park fairfield, bike racks for sale, sherando lake campground, days inn williamsburg va, sparks marina, crescent beach club bayville, bobby flay ribs, kettle moraine golf course, cheap bmx, gsga.org, smaff, jabsco marine toilet, ri things to do, hotels nc, sea chambers ogunquit, sandy pines michigan, four mile ranch, motel pigeon forge tn, nb canada, motel fish, mapmyride.com, conesus lake ny, pomo canyon campground, golf course madison wi, hotels ca, rent camping equipment, erie canal cruises, car rental boston ma, cornerstone gold, bike trip planner, 26101 magic mountain parkway valencia, ca 91355, embroidery floss bracelets, great plains zoo sioux falls, 26101 magic mountain parkway, raceway golf course, village by the sea wall maine, cayuga lake cruises, blue crab info, red reef park boca, hot deals hotels, st louis mo attractions, ocean city nj events, hotel york maine, tent pole technologies, golf course louisville ky, creekstone inn pigeon forge tn, sandstone amphitheater, produce stand, ohsweken, floss friendchip bracelets, aston oaks golf course, loans for, seattle airport car rental, lake almanor ca, inn newport ri, pfd types, chutes du niagara, lake chelan golf course, shining sea bike path, bike map nyc, parques de diversion, bald head island map, beechridge motor speedway, broad run golf course, cheap bmx bikes, rocktide inn boothbay harbor, juday creek golf course

West Search Terms Identified by Google Correlate™	Symptom and Ixodid-vector Terms added for Each Region
zucchini blossom, zucchini flower, pick blueberries, blueberry farm, blueberry picking, blarney island, pick your own blueberries, blueberry patch, oak street beach chicago, state park mi, freezing peas, waterworld denver, college bedding, cascade lake, lake water park, summer sizzler, lower huron, lake in nj, chautauqua ny, beach in nj, blueberry hill farm, freezing squash, beach in ny, lake beaches, u pick blueberries, wolf river campground, nj water temperature, vine borers, u pick berries, presque isle erie, sackets, warner center park, oak street beach, memorial beach, music in the park, raspberry cobbler, nara park acton, wi beaches, free concerts, free outdoor concerts, beach in ri, calf pasture beach, squash flowers, berry picking, gretna lake, concert in park, hotels near dornery park, concert in the park, caribfest, free concert, jacob riis beach, water west, music under the stars, wild water west, roseville water park, mercer county fair, fried squash, sand castle contest, riverview campground, bandshell, broomfield bay, buffalo state park, lake quassy, sprinkler park, water tubing, old forge camping, beach ct, nock a mixon, beach in ct, trails end camp, market fest, watermelon soup, movies in park, huron metro park, soak city, sunken meadow beach, calf pasture, nara park, movie in park, higgins lake state park, how to freeze blueberries, ny beach, wpba baseball, ma tide, coupons for water, wild water, water kingdom, hotels near worlds of fun, river tube, renting kayaks, the blueberry patch, mt adams, freeze blueberries, davis park ferry schedule, echo lake campground, south haven hotels, outdoor movies, outdoor water park, movies in the park, wild water kingdom	tick, black tick, lyme, lyme disease, rash ,bullseye rash, bell's palsy, facial paralysis, side of face paralyzed, knee pain, swollen knees, swollen joint, swollen joints, joint pain, fevre, tired, deer tick, black-legged tick, black legged tick, black leg tick
