# Supplementary material for: Inclusion of environmentally themed search terms improves Elastic net regression nowcasts of regional Lyme disease rates
Source: PLoS One. 2022 Mar 10;17(3):e0251165. doi: 10.1371/journal.pone.0251165 (PMC8912246; doi:10.1371/journal.pone.0251165)
Supplement: S2 Table — Pearson Correlations values were calculated between each term monthly proportional search data and corresponding Lyme disease rates for each term and region. (PDF) [file pone.0251165.s002.pdf]

S2 Table Bivariate Correlations of each search term to the regional Lyme disease rate

Northeast		Midwest		Southeast		Southwest		West	
**Search Term**	**Corr. Value**	**Search Term**	**Corr. Value**	**Search Term**	**Corr. Value**	**Search Term**	**Corr. Value**	**Search Term**	**Corr. Value**
july calendar	0.89**	kings island discount	0.90**	intex	0.84**	loans for	0.61**	movies in park	0.84**
free concerts	0.88**	beaches in michigan	0.90**	cloudy pool	0.84**	hotels ca	0.55**	movies in the park	0.83**
movies under the stars	0.87**	festivals milwaukee	0.89**	summer things	0.81**	ca water	0.55**	movie in park	0.82**
lyme	0.85**	easy summer recipes	0.88**	baking soda pool	0.80**	deer tick	0.45*	concert in the park	0.80**
summer recipe	0.85**	lake beaches	0.88**	green pool	0.80**	moon bay ca	0.44*	berry picking	0.80**
lyme disease	0.85**	motel wisconsin dells	0.87**	alabama water park	0.79**	half moon bay ca	0.40*	blueberry farm	0.79**
little league all stars	0.85**	blueberry farm	0.85**	cloudy pool water	0.79**	make string bracelets	0.40*	concert in park	0.79**
necbl	0.84**	summer desserts	0.85**	summer things to do	0.79**	rash	0.39*	blueberry picking	0.78**
berry picking	0.83**	movies in the park	0.85**	blue bayou in baton rouge	0.77**	tick	0.38*	outdoor movies	0.77**
alive at five	0.83**	watermelon recipe	0.84**	springtails	0.75**	how to make string bracelets	0.38*	lake water park	0.76**
prospect park bandshell	0.82**	summer appetizers	0.84**	alabama water parks	0.73**	string bracelets	0.36*	broomfield bay	0.75**
freezer jam	0.82**	dorm bedding	0.84**	point mallard	0.73**	recipes on the grill	0.34*	waterworld denver	0.75**
harwich mariners	0.81**	wild water west	0.84**	park pools	0.72**	pigeon forge hotels	0.33*	u pick berries	0.72**
brooklyn concerts	0.79**	summer dessert	0.83**	swampdogs	0.72**	lyme disease	0.31*	free concerts	0.72**
concerts brooklyn	0.77**	49 drive in	0.83**	clear pool water	0.72**	ri things to do	0.31*	warner center park	0.72**

cherry pitter	0.77**	drive in ohio	0.82**	tick	0.71**	seattle airport car rental	0.30*	lake beaches	0.72**
gatemen	0.77**	free concerts	0.82**	haier air	0.71**	hotels nc	0.29*	music in the park	0.70**
central park free concerts	0.77**	harvesting garlic	0.80**	summer books	0.71**	lyme	0.26*	soak city	0.69**
free concerts in nyc	0.76**	river water park	0.80**	intex pool cover	0.70**	joint pain	0.24*	college bedding	0.67**
keene swamp bats	0.76**	music in the park	0.80**	intex pumps	0.70**	tired	0.24*	u pick blueberries	0.66**
freedom fest	0.76**	coney island cincinnati	0.79**	green pool water	0.70**	knee pain	0.24*	river tube	0.65**
fresh cherry recipes	0.76**	outdoor water parks	0.79**	swim shirts	0.69**	black tick	0.23*	coupons for water	0.64**
summer in the park	0.75**	water west	0.79**	lyme disease	0.69**	world rv	0.22*	roseville water park	0.63**
concert in park	0.75**	family aquatic center	0.79**	lyme	0.68**	sandstone amphitheater	0.21*	wild water	0.63**
fresh cherry	0.74**	wild water	0.78**	banzai water	0.68**	cave of the winds colorado springs	0.20*	free concert	0.59**
swamp bats	0.74**	blueberry patch	0.77**	blue bayou	0.67**	cheap bmx bikes	0.20*	summer sizzler	0.59**
nycbl	0.74**	sprinkler park	0.77**	amc free movies	0.67**	cheap bmx	0.17	raspberry cobbler	0.59**
newport gulls	0.73**	hotels near worlds of fun	0.76**	bullseye rash	0.67**	bullseye rash	0.15	cascade lake	0.58**
bullseye rash	0.73**	melody 49 drive in	0.76**	auburn water park	0.63**	swollen knee	0.14	blueberry hill farm	0.57**
bourne braves	0.73**	funplex omaha	0.76**	east cobb baseball	0.62**	swollen knees	0.14	mt adams	0.57**
blueberry farms	0.71**	centennial beach	0.76**	rash	0.61**	ohsweken	0.10	how to freeze blueberries	0.56**
summer sizzler	0.71**	beaches in ohio	0.75**	wilmington sharks	0.61**	lake almanor ca	0.09	echo lake campground	0.55**
freezing cherries	0.70**	lyme disease	0.73**	valley beach	0.60**	black legged tick	0.09	rash	0.54**

wild raspberries	0.70**	what do baby toads eat	0.73**	coastal plains league	0.59**	cataratas del niagara	0.06	zucchini flower	0.52**
tomato plant problems	0.69**	harvesting basil	0.72**	baler parts	0.59**	produce stand	0.04	outdoor water park	0.51**
summer run	0.69**	lyme	0.71**	deer tick	0.59**	swollen joints	0.04	water tubing	0.51**
swimming parks	0.69**	worlds of fun discount	0.71**	top summer songs	0.57**	carpinteria	0.01	fried squash	0.50**
falmouth commodores	0.68**	pick blueberries	0.71**	summer movie camp	0.57**	facial paralysis	-0.02	tick	0.45*
fresh cherry pie	0.67**	beach chicago	0.71**	pool rope	0.57**	swollen joint	-0.04	bullseye rash	0.44*
pick your own blueberries	0.67**	homemade ice cream recipes	0.70**	pa pool	0.57**	fever	-0.05	squash flowers	0.43*
rash	0.67**	splash country	0.70**	elite camp	0.56**	nb canada	-0.06	freeze blueberries	0.42*
fourth of july cakes	0.67**	dollywood splash country	0.69**	quick set pool	0.55**	bell's palsy	-0.06	lyme	0.42*
picking blueberries	0.66**	island water	0.69**	amc free	0.54**			bandshell	0.40*
blossom rot	0.66**	luverne drive in	0.69**	martinsville mustangs	0.53**			market fest	0.39*
collegiate baseball league	0.66**	fried squash	0.69**	wood bat	0.52**			water kingdom	0.38*
all stars baseball	0.66**	island water park	0.69**	pool times	0.52**			blueberry patch	0.38*
rose tree park	0.64**	splashdown water park	0.65**	baler	0.51**			music under the stars	0.36*
harvesting basil	0.64**	bay water park	0.64**	bag toss	0.50*			lyme disease	0.34*
summer concerts in the park	0.63**	grilled squash	0.63**	hay rake	0.48*			ny beach	0.34*
deer tick	0.60**	campground colorado	0.63**	black tick	0.47*			water west	0.33*

symphony in the park	0.57**	rash	0.63**	summer family fun	0.42*
akron racers	0.56**	buffalo state park	0.62**	free summer movie	0.37*
tick	0.55**	pikes peak cog railway	0.62**	easy camp	0.33*
valley league baseball	0.54**	marketfest	0.60**	us club	0.31*
free outdoor concerts	0.51**	summer sizzler	0.59**	summertime songs	0.31*
coastal plains league	0.50**	bullseye rash	0.59**	swollen knee	0.28*
baby toads	0.48*	tick	0.58**	cast cover	0.27*
pick your own berries	0.48*	deer tick	0.57**	black legged tick	0.25*
pitting cherries	0.47*	pool cooler	0.54**	fun days	0.25*
cherry recipes	0.44*	venture river	0.54**	joint pain	0.24*
freedom run	0.42*	wolf river campground	0.54**	swollen joint	0.22*
white water six flags	0.39*	music under the stars	0.53**	tired	0.20*
watertown wizards	0.38*	summer soup	0.53**	knee pain	0.18*
joint pain	0.35*	cooling bandana	0.49**	nycbl	0.17*
lake fest	0.35*	valley beach	0.43	pool supply stores	0.14
black tick	0.34*	black tick	0.41**	swollen joints	0.12
knee pain	0.33*	wetlands water park	0.40*	swollen knees	0.05
milwaukee festival	0.32*	joint pain	0.40*	facial paralysis	-0.07

sand castle contest	0.33*
beach ct	0.33*
sprinkler park	0.33*
beach in ny	0.31*
free outdoor concerts	0.30*
deer tick	0.29*
wild water kingdom	0.29*
beach in ct	0.29*
watermelon soup	0.28*
wild water west	0.28*
tired	0.27*
beach in nj	0.26*
knee pain	0.25*
joint pain	0.25*
buffalo state park	0.24*
the blueberry patch	0.24*
swollen knee	0.20*
oak street beach	0.18*

passing league	0.30*
church festivals	0.30*
water park ga	0.25*
black legged tick	0.25*
freezing squash	0.24*
water park atlanta	0.24*
swollen joint	0.23*
swollen knee	0.20*
big surf	0.18*
swollen knees	0.14
tired	0.14
alaska baseball league	0.08
bell's palsy	0.07
swollen joints	0.06
facial paralysis	0.04
marketfest	-0.06
fever	-0.28*
bible school songs	*NA*
vbs games	*NA*

swollen knee	0.34*
waterworld concord	0.34*
watermelon drinks	0.33*
festival colorado	0.30*
knee pain	0.30*
waterworld denver	0.26*
black legged tick	0.25*
sherwood island state park	0.24*
tired	0.21*
swollen knees	0.20*
little buffalo state park	0.17
fantasy lake	0.14
swollen joint	0.13
swollen joints	0.03
bell's palsy	-0.02
facial paralysis	-0.02
fever	-0.25*
river float trips	*NA*

fever	-0.10
bell's palsy	-0.14
alive at five	*NA*
asheboro copperheads	*NA*
danville dans	*NA*
diamond devils	*NA*
fayetteville swampdogs	*NA*
free summer kids movies	*NA*
herndon braves	*NA*
palmetto falls	*NA*
wilson tobs	*NA*
wood bat tournament	*NA*

swollen joint	0.16
memorial beach	0.16
state park mi	0.15
black legged tick	0.10
nara park	0.08
blarney island	0.08
lake in nj	0.05
chautauqua ny	0.02
swollen knees	-0.01
swollen joints	-0.03
black tick	-0.04
sackets	-0.12
bell's palsy	-0.14
facial paralysis	-0.14
fever	-0.17

$* p < 0.05$. $** p << 0.05$
