# Supplementary material for: Inclusion of environmentally themed search terms improves Elastic net regression nowcasts of regional Lyme disease rates
Source: PLoS One. 2022 Mar 10;17(3):e0251165. doi: 10.1371/journal.pone.0251165 (PMC8912246; doi:10.1371/journal.pone.0251165)
Supplement: S3 Table — (PDF) [file pone.0251165.s003.pdf]

S3 Table Predictions from symptoms and vector terms only models produce accurate predictions with low error

	Northeast		Midwest		Southeast		Southwest		West	
	M1	M2	M1	M2	M1	M2	M1	M2	M1	M2
**α, ƛ**	0.47, 0.62	0.47, 0.60	0.34, 0.20	0.33, 0.20	0.28, 0.07	0.29, 0.07	0.14, 0.01	0.11, 0.01	0.1, 0.01	0.1, 0.01
**Training**										
RMSE	1.32	1.32	0.36	0.36	0.11	0.11	0.01	0.01	0.01	0.01
MAE	0.89	0.89	0.21	0.21	0.07	0.07	0.01	0.01	0.01	0.01
R^2	0.77	0.77	0.65	0.65	0.67	0.67	0.32	0.32	0.51	0.50
**Validation**										
RMSE	1.50	1.50	0.38	0.38	0.11	0.11	0.01	0.01	0.01	0.01
MAE	1.01	1.01	0.25	0.25	0.07	0.07	0.01	0.01	0.01	0.01
R^2	0.71	0.71	0.59	0.59	0.69	0.69	0.38	0.38	0.29	0.29
**Out of Sample**										
RMSE	1.65	1.65	0.43	0.43	0.14	0.14	0.01	0.01	0.01	0.01
MAE	1.38	1.38	0.34	0.34	0.10	0.10	0.01	0.01	0.01	0.01
R^2	0.79	0.79	0.76	0.76	0.82	0.82	0.37	0.37	0.64	0.63
