# Supplementary material for: Inclusion of environmentally themed search terms improves Elastic net regression nowcasts of regional Lyme disease rates
Source: PLoS One. 2022 Mar 10;17(3):e0251165. doi: 10.1371/journal.pone.0251165 (PMC8912246; doi:10.1371/journal.pone.0251165)
Supplement: S4 Table — (PDF) [file pone.0251165.s004.pdf]

S4 Table Predictions form full list models produce highly accurate predictions with low error

	**Northeast**		**Midwest**		**Southeast**		**Southwest**		**West**	
	M1	M2	M1	M2	M1	M2	M1	M2	M1	M2
**α, ƛ**	0.1, 0.93	0.1, 0.85	0.93, 0.00	0.93, 0.00	0.1, 0.07	0.1, 0.07	0.1, 0.01	0.1, 0.01	0.1, 0.00	0.1, 0.00
**Training**										
RMSE	0.67	0.66	0.12	0.12	0.06	0.06	0.01	0.01	0.01	0.01
MAE	0.47	0.46	0.08	0.09	0.04	0.04	0.01	0.01	0.00	0.00
R^2	0.94	0.94	0.96	0.95	0.91	0.91	0.56	0.56	0.84	0.84
**Validation**										
RMSE	1.00	0.99	0.23	0.23	0.08	0.08	0.01	0.01	0.01	0.01
MAE	0.62	0.62	0.14	0.14	0.05	0.05	0.01	0.01	0.01	0.01
R^2	0.87	0.87	0.85	0.85	0.84	0.84	0.44	0.44	0.70	0.70
**Out of Sample**										
RMSE	0.75	0.74	0.28	0.29	0.14	0.14	0.01	0.01	0.01	0.01
MAE	0.53	0.52	0.17	0.17	0.09	0.09	0.01	0.01	0.01	0.01
R^2	0.97	0.97	0.94	0.94	0.91	0.91	0.45	0.45	0.82	0.82
