# Supplementary material for: Inclusion of environmentally themed search terms improves Elastic net regression nowcasts of regional Lyme disease rates
Source: PLoS One. 2022 Mar 10;17(3):e0251165. doi: 10.1371/journal.pone.0251165 (PMC8912246; doi:10.1371/journal.pone.0251165)
Supplement: Attachment — Submitted filename: PLOSOne_response to ReviewerComments final.docx [file pone.0251165.s006.docx]

We thank Dr. Telford, the editor, and our reviewers for their thoughtful comments. We agree that the rigorous context of Mavragani and Ochoa, 2019, and in a new summary table, containing what they emphasize as the four key components for this type of computational epidemiology adds great strength to this method and specifically our manuscript. We have added this summary table at line 293 containing the five regions, the top 10, focused, keywords (so categories not needed) over the critical training period, 2004-2012.

Our responses will appear below in Garamond.

a) Please clarify the sources of funding (financial or material support) for your study. List the grants or organizations that supported your study, including funding received from your institution.

There were no sources of funding for this research

b) State what role the funders took in the study. If the funders had no role in your study, please state: “The funders had no role in study design, data collection and analysis, decision to publish, or preparation of the manuscript.”

The funders had no role in study design, data collection and analysis, decision to

publish, or preparation of the manuscript.

c) If any authors received a salary from any of your funders, please state which authors and which funders.

No authors received a salary from any of our funders.

d) If you did not receive any funding for this study, please state: “The authors received no specific funding for this work.”

The authors received no specific funding for this work.

5. Review Comments to the Author

Reviewer #1: This manuscript proposes a method to forecast Lyme disease incidence using regressions methods with Google search history data. I believe that the application of machine learning/Big Data methods is misguided for ecologically complex phenomena like the incidence tick-borne diseases. This manuscript does not provide any compelling evidence for the contribution of these techniques to the understanding of Lyme disease incidence. As an exquisitely seasonal process, Lyme disease perpetuation and zoonotic transmission will be powerfully correlated to Google search terms. The same would be true for indicators of “nice weather” (not obtained from Google!).

We appreciate the considerations of this reviewer and completely agree that the ecological system of *Borrelia* is complex. This exercise was to determine how well these computational methods could reproduce and “nowcast” trends of reported Lyme Disease, and surprisingly it did quite well and found trends even if the weather was not nice… like the fall adult *Ixodes* transmission “bump”. That is why we feel this should be reported through this work.

Reviewer #2: General comments
This is a paper on inclusion of environmentally themed search terms improved Elastic Net regression nowcasts of regional Lyme disease rates. I have some comments on your manuscript.

Specific comments
1. Abstract and Introduction: ”…with 95% of human cases occurring…” Please provide the absolute numbers of cases (n/N), showing where this percentage is coming from.

We have now included the total number of confirmed positive cases (n) from northeast and upper Midwest states and the total number of confirmed positive cases for the United

states (N).

2. Introduction: “CDC”. Please write this out when mentioned for the first time in the main text.

We have included the full name (Center for Disease Control and Prevention) of the CDC

before its first mention in the introduction

3. Introduction: “LASSO”. Please write this out when mentioned for the first in the text.

We have included the full name (Least Absolute Shrinkage and Selection Operator) of

the LASSO before its first mention in the introduction.

4. Material and Methods: “erythema migrans”. You may consider briefly describing what this is, you may put this description in the parentheses, for example.

We have added “bullseye rash” as a parenthetical comment in this section.

5. Please check up the capital letters concerning Google, United States, Table, for example. Also, some words are lowercased instead of capital letters.

We have made edits throughout the manuscript to maintain consistency of capitalization and apologize for these errors.

6. Both abbreviations are used: “US” and U.S.”. Please consider choosing one of them.

We have edited the manuscript to maintain consistency of US throughout.

7. Figure 7: If you use color lines in the figures, please tell the readers which color indicates which line.

We have included text into the legend of Figure 7 to make clear that the black lines are regional incidence and the colored lines are model predictions.

8. Please check up the reference list concerning the links and make sure that they are updated.

We have updated the reference list to ensure that the all links are active and

functioning.

Reviewer #3: This is an interesting approach in modeling Lyme Disease with Google Trends data. However, there are some issues that need to be addressed before this manuscript can be reconsidered for publication.

1. The authors mention that “High correlation was determined when the correlation value was greater than 0.8, moderate if correlation value was between 0.5 and 0.8, and poor when less than 0.5”. Shouldn’t the significance of a correlation be measured by, for example, the p-values (or CIs)? Also, “high” and “moderate” should be defined (I assume the authors mean that high is p<0.01 and that moderate is p<0.05; however, a correlation with a p-value less than .05 is considered quite strong).

We thank the reviewer for this comment, and indicated when p-values were significant

in Supplemental Table 2 to accompany the correlation values. We feel that reporting the

correlation value directly is important, as this measures the strength of the linear

relationship directly. P-values (and by extension confidence intervals) measure the

strength of evidence for the presence of nonzero correlation, but do not give any

indication of the strength of correlation itself. In a large sample with multiple comparisons, one could obtain very strong evidence for nonzero correlation when the linear relationship is actually quite weak, while in a small sample a high correlation might not reach significance.

2. There is no description of the Google Trends data selection criteria and collection procedure. This is an important drawback of this manuscript. All methodology steps should be reported in detail (e.g., period, region, category, web search, use of quotes for keywords with more than one word, individual searches, comparisons, etc.).

We have added to the methods section to make this clear. We state in the methods that we use the terms identified via Google Correlate to collect search hit data on. We have added more language to make it clear that terms identified from Google Correlate were inputted into Google Trends unaltered to collect search term hit data for each region. I also included text to make it clear that gtrendsR is an R interface for Google Trends that allows for an automated process of collecting search term data.

3. This is an information epidemiology (infodemiology) study. I suggest that the authors study the relevant literature in order to gain insight and enhance their literature review. An introductory paragraph could be added in the Introduction Section.

We have included a paragraph, lines 93-102, to the introduction outlining infodemiology and its use in predicting disease and better informing the general public about health-related outcomes.

4. The analysis (data collection) was conducted in September 2019, considering data up to December 2018. It is now 2021, and there are two more years’ data available. I believe it would add to the value of this manuscript if the analysis was updated.

We appreciate and understand the reviewers concern for having recent data for publication. However, the authors’ intention of this work is to show that value of including environmentally related features when nowcasting with Google Search terms, which does not require all recent data. This manuscript highlights the importance of considering environmental factors when creating prediction models for vector borne diseases. To this end, the models have also been posted on Eric Kontowicz’s Github (<https://github.com/ekontowicz/Lyme-disease-Elastic-Net-regression-Nowcasting>) for further use by other researchers and additional updates. Lastly, given the time it takes to have rigorous peer review, particularly during this COVID-19 pandemic these findings will lag all surveillance data available.
